# Diagnostic, Prognostic, and Predictive Molecular Biomarkers and the Utility of Molecular Imaging in Common Gastrointestinal Tumors

**DOI:** 10.1155/2015/890805

**Published:** 2015-11-04

**Authors:** Michael O. Idowu, Jennifer Laudadio, Kathryn Rizzo

**Affiliations:** ^1^VCU Medical Center, MCV Campus, P.O. Box 980662, Richmond, VA 23298-0662, USA; ^2^Department of Pathology, University of Arkansas for Medical Sciences, 4301 W. Markham Street, No. 517, Little Rock, AR 72205, USA

The exponential increase in the use of molecular biomarkers as diagnostic, prognostic, and predictive aids in the management of cancer patients highlights the increasing importance of molecular biology in oncology. The clinical utility of some molecular biomarkers like* KRAS* (Kirsten rat sarcoma viral oncogene homolog),* BRAF* (B-Raf protooncogene, serine/threonine kinase),* PIK3CA* (phosphatidylinositol-4,5-bisphosphate 3-kinase, catalytic subunit alpha),* KIT* (commonly known as cKit) (v-kit Hardy-Zuckerman 4 feline sarcoma viral oncogene homolog),* ERBB2* (commonly known as HER2) (erb-b2 receptor tyrosine kinase 2), and* EGFR* (epidermal growth factor receptor) among others has been validated in gastrointestinal and pancreatobiliary tumors. However, the clinical utility of some of molecular biomarkers is still being investigated and validated. Although technically not a “molecular biomarker,” the utility of “molecular imaging” is being elucidated.

This special issue covers some of the biomarkers currently in current clinical use and others being investigated, including the following: (i)* MMP14* (previously known as* MT1-MMP*) (matrix metallopeptidase 14 (membrane-inserted) or previously known asmatrix metalloproteinase 14 (membrane-inserted)) and role in colorectal cancer, potential utility being described in other cancers [1–4], (ii)* SLC6A14* (solute carrier family 6 (amino acid transporter), member 14) and potential role in pancreatic cancer, potential utility in other cancers being described [5–7], (iii) molecular profiling [8–14] of tumors to detect potentially actionable mutation or variant in pancreatic cancers, and (iv) potential utility of Raman spectroscopy in evaluation of gastrointestinal lesions. Potential utility of this technology has been described in other tumors [15–20].

*Michael O. Idowu* *Jennifer Laudadio* *Kathryn Rizzo*
